# Label-free molecular profiling of cancer using Raman spectroscopy: from fundamentals to clinical applications

**DOI:** 10.3389/fonc.2026.1822181

**Published:** 2026-07-03

**Authors:** Minglong Li, Dongjie Yang, Tong Liu, Shiyin Liu, Wenqi Sun, Ping Cai, Yanping Shen, Weimin Zhang, Guye Lu, Yuan Weng, Haifeng Wang, Xiaoyu Zhao, Jinyou Li

**Affiliations:** 1 Department of Thoracic Surgery, Affiliated Hospital of Jiangnan University, Wuxi, China; 2 Wuxi Medical College of Jiangnan University, Wuxi, China; 3 Department of Pathology, Affiliated Hospital of Jiangnan University, Wuxi, China; 4 Department of Pulmonary Medicine, Wuxi People’s Hospital, Wuxi, China; 5 Department of Thoracic Surgery, Shanghai Pulmonary Hospital, Shanghai, China; 6 SuperVision Medicine Co., Ltd., Wuxi, China

**Keywords:** cancer diagnostics, quantitative molecular imaging, Raman imaging, Raman spectroscopy, real-time histopathology

## Abstract

Early cancer detection and individualized therapy represent two of the foremost challenges in contemporary oncology. Raman spectroscopy, a rapid and label-free technique based on molecular vibrations, has emerged as a powerful tool with significant potential to address these needs. It offers unique capabilities for early cancer detection, real-time histopathological evaluation, tumor classification, and intraoperative diagnosis. This article reviews the fundamental principles of spontaneous Raman spectroscopy and highlights recent advances in enhanced Raman techniques, including coherent anti-Stokes Raman scattering (CARS) and stimulated Raman scattering (SRS). We examine their applications in diagnosing cancers of the lung, liver, colorectum, gastrointestinal tract, and other solid tumors, with a focus on early cancer detection, real-time histopathological evaluation, and label-free tissue characterization. Furthermore, we explore the use of Raman spectroscopy in studying tumor metabolic profiles and the tumor microenvironment, such as extracellular matrix (ECM) profiling, enabling new insights into metabolic imaging and vibrational phenotyping. The review also addresses key technical and translational challenges in Raman-based cancer diagnosis—including signal enhancement, spectral unmixing, instrument miniaturization, and clinical interpretability. Finally, we outline future directions for developing integrated, multimodal diagnostic platforms that combine vibrational spectroscopy with other optical methods. These advances hold great promise for achieving real-time, precise, and personalized cancer management.

## 1 Introduction

Despite significant advances in cancer diagnosis, diagnostic errors and delays remain prevalent and detrimental in contemporary clinical practice (1). These challenges are pervasive across cancer types and clinical settings. Persistent issues such as tumor heterogeneity, sampling limitations, and methodological constraints continue to impede early detection and reliable diagnosis, particularly in contexts reliant on tissue biopsy (2).

For instance, in the lung cancer screening population, approximately 15% of cancers in organized programs are attributed to false-negative low-dose CT examinations (3). In breast cancer screening, a residual risk of missed diagnoses persists despite improvements offered by digital breast tomosynthesis (4). Autopsy syntheses among modern ICU populations have consistently reported high rates of major diagnostic discrepancies, including missed malignant disease, highlighting a significant diagnostic pathway gap (5).

**Figure 1:**
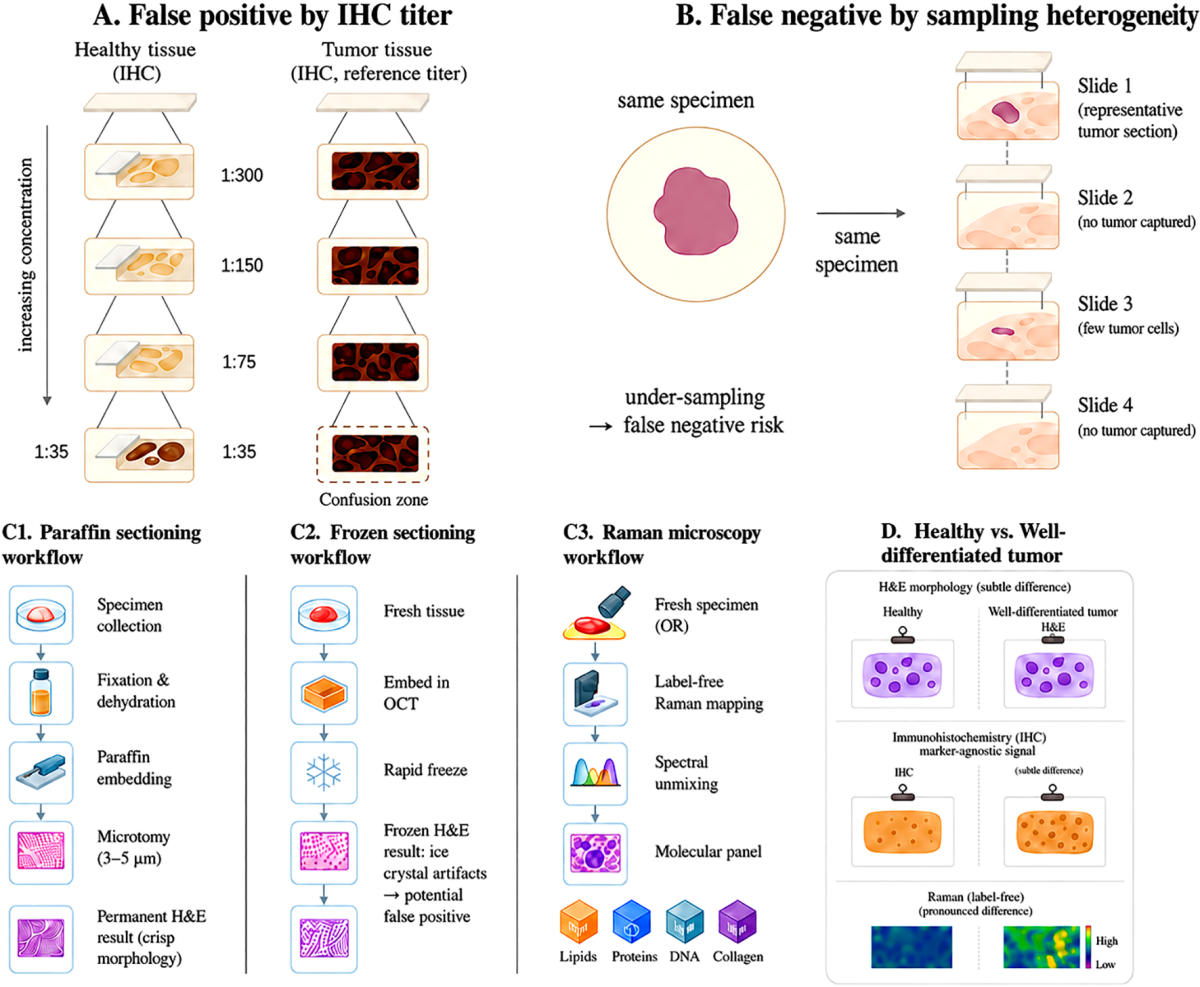
Common problems in the current histopathological workflow and the application prospects of Raman microscopy. **(A)** False positive risk related to immunohistochemistry (IHC). Excessive antibody concentration can lead to over-staining, causing healthy tissues to be mistakenly classified as malignant tissues. **(B)** False negative risk caused by sampling heterogeneity. In the same tumor, different sections may produce inconsistent results: Sections 1 and 3 show the presence of a tumor, while sections 2 and 4 appear normal. (C1) Standard paraffin sectioning procedure, which is the traditional gold standard for confirming pathological diagnosis. (C2) The cryo-sectioning process is currently used for intraoperative margin assessment, but it is limited by sectioning quality, sampling errors, and the subjectivity of interpretation. (C3) Raman microscopy workflow, a label-free and molecular-specific method that enables real-time tissue characterization without dyes or contrast agents. **(D)** Representative Raman test results show spectral differences between healthy tissues and well-differentiated tumors, demonstrating its ability to distinguish at the molecular level.

Similar pitfalls are observed in histopathological workflows. As illustrated in **Figure 1A**, excessive immunohistochemistry (IHC) titers can generate false-positive diagnoses, whereby healthy tissues are misclassified as malignant. Conversely, sampling heterogeneity, shown in **Figure 1B**, can result in false negatives: in the same tumor, some slides may reveal malignancy (slides 1 and 3), whereas others appear normal (slides 2 and 4). Such discrepancies highlight the intrinsic risks of both false-benign and false-malignant calls.

Beyond initial diagnosis, the accurate determination of tumor margins during surgery presents another major challenge. In breast-conserving surgery, incomplete resection resulting in positive or close margins remains a strong predictor of local and distant recurrence, as supported by a prospectively registered BMJ meta-analysis (6). Conversely, excessive resection is associated with significant complications and long-term disability (7, 8). Uncertainty regarding intraoperative margins complicates real-time surgical decision-making, leading to both undertreatment (e.g., residual tumor) and overtreatment (e.g., unnecessary re-excision). Consequently, there is a strong demand for real-time margin assessment tools (9).

The current standard of care relies on frozen-section pathology (**Figure 1C2**), which is rapid but limited by section quality, sampling error, and interpretive subjectivity (10, 11). Discrepancies often arise between frozen and permanent section findings, sometimes leaving residual tumor behind or prompting unnecessary tissue removal (9). Diagnostic accuracy is also highly operator-dependent and can be impaired by freezing artifacts and suboptimal nuclear/cytoplasmic detail (10). In equivocal cases, definitive classification usually requires immunohistochemistry post-surgery (**Figure 1C1**) rather than intraoperatively (12). Such limitations may delay or misdirect treatment decisions (13).

In response to these challenges, non-invasive, real-time, and molecularly specific technologies are urgently needed to guide preoperative planning and provide intraoperative guidance (14). Raman microscopy, as shown in **Figure 1**C3, offers a promising alternative. Raman spectroscopy is a label-free optical technique that utilizes inelastic photon scattering to probe the vibration of molecular bonds, producing biochemical “fingerprints” that enable sensitive differentiation between benign and malignant tissues (15).

In oncology, Raman-based approaches have demonstrated significant diagnostic utility across a range of applications, from rapid tissue classification to intraoperative assessment (15). By capturing intrinsic vibrational fingerprints derived from nucleic acids, proteins, and lipids, Raman spectroscopy enables discrimination between benign and malignant tissues without the need for exogenous dyes or contrast agents. This capability supports critical clinical tasks including cancer detection, subtype classification, and tumor-margin delineation (15). An example of such discrimination between healthy and well-differentiated tumor tissue is shown in **Figure 1D**.

Recent advances, such as hyperspectral penalized reference matching stimulated Raman scattering (PRM-SRS), have further enhanced chemical specificity and processing speed, extending the scope of *in vivo* and intraoperative diagnostic applications (16). However, two major challenges must be overcome for full clinical translation: robust spectral unmixing and the establishment of standardized data processing pipelines (16). Addressing these issues is essential for the seamless integration of Raman-based diagnostics into existing clinical oncology workflows (14).

## 2 The basic principles of Raman spectroscopy

**Figure 2:**
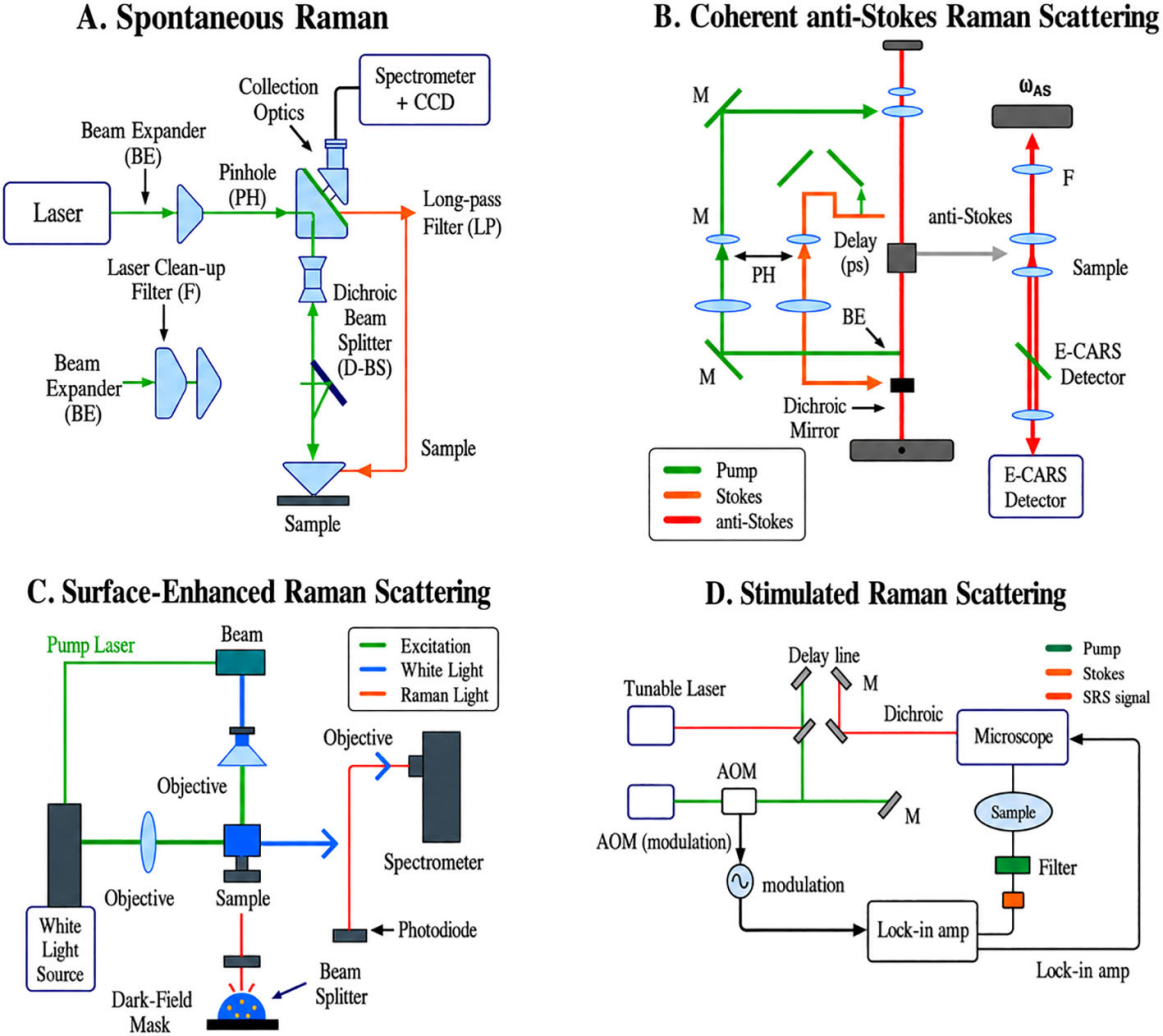
Schematic diagram of the optical path for the main Raman spectral modes. **(A)** Spontaneous Raman scattering: A single laser excitation triggers weak inelastic scattering from the sample, and the collected Raman signal is directed to a spectrometer and CCD detector for spectral analysis. **(B)** Coherent anti-Stokes Raman scattering (CARS): Synchronized pump and Stokes beams excite molecular vibrations and generate a blue-shifted anti-Stokes signal (ωAS), which can be collected in forward or epi-detection geometry. **(C)** Surface-enhanced Raman scattering (SERS): A representative SERS detection layout is shown. Raman signals are enhanced when target molecules are close to plasmonic surfaces or nanostructures, mainly through electromagnetic enhancement and, to a lesser extent, chemical enhancement, enabling sensitive detection of trace molecules. **(D)** Stimulated Raman scattering (SRS): Pump and Stokes beams are modulated and detected with a lock-in amplifier to generate high-speed Raman contrast for imaging. BE, beam expander; BS, beam splitter; PH, pinhole; LP, long-pass filter; D-BS, dichroic beam splitter; M, mirror; F, filter; CCD, charge-coupled device; AOM, acousto-optic modulator; PMT, photomultiplier tube; ωAS, anti-Stokes signal frequency; E-CARS, epi-detected CARS; Lock-in amp, lock-in amplifier.

Raman-based techniques decode molecular vibrational characteristics through inelastic light scattering and can provide non-invasive and chemically specific analysis of biological samples without exogenous labeling (17). As shown in **Figure 2**, the main Raman modalities covered here include spontaneous Raman spectroscopy/microscopy (**Figure 2A**), coherent anti-Stokes Raman scattering (CARS; **Figure 2B**), surface-enhanced Raman scattering (SERS; **Figure 2C**), and stimulated Raman scattering (SRS; **Figure 2D**). These modalities differ in signal strength, spectral coverage, imaging speed, spatial resolution, sensitivity, and biochemical specificity (18, 19).

Because these modalities can be used in different acquisition formats, several related terms should be distinguished. Raman spectroscopy generally refers to spectral acquisition from a point, a line, or a selected region of a sample, providing molecular vibrational fingerprints. Raman microscopy describes Raman spectral measurements performed under a microscope, usually for cells or small tissue regions. When spectra are collected across a defined area and reconstructed into molecular distribution maps, the term Raman imaging is used. The term Raman-based approaches is used as a broader description that includes these Raman modalities as well as related clinical and computational implementations, such as fiber-optic Raman probes, hyperspectral Raman platforms, and Raman methods combined with machine learning or artificial intelligence.

### 2.1 Spontaneous Raman imaging

Spontaneous Raman microscopy (**Figure 2A**) captures the inherent vibration signal through a single laser excitation and can achieve label-free molecular characterization over a wide spectral range (17). Its ability to cover the fingerprint region (400–1800 cm^-^¹) and the high wavenumber region (2800–3200 cm^-^¹) enables the acquisition of rich molecular information, ranging from nucleic acids and proteins to extracellular matrix (ECM) components (20, 21). The spectral resolution of modern systems can reach 1–3 cm^-^¹, which is sufficient to distinguish subtle spectral differences in complex biological environments (22). This wide-spectrum coverage enables it to directly distinguish nucleic acids, lipids, proteins, carbohydrates and ECM components in tissues (23, 24). Raman spectral projection tomography (RSPT) is a significant advancement that has enabled three-dimensional volumetric molecular imaging in living tissues (25).

Unlike traditional methods based on decellularization that disrupt spatial structure, spontaneous Raman can preserve the natural molecular and structural background of ECM *in situ* (24, 26). However, its application is constrained by inherent limitations: spectral overlap and crowding between biomolecular peaks often make it difficult to identify them clearly, especially for ECM proteins with similar structures (27). Emerging computational methods, such as physically informed autoencoders for hyperspectral unmixing, have shown potential in overcoming these challenges (28). In addition, the integration with multimodal platforms such as hyperspectral SRS (**Figure 2D**) can provide complementary high-speed molecular imaging (29, 30).

### 2.2 Coherent Raman scattering: CARS and SRS

Coherent Raman scattering techniques, including coherent anti-Stokes Raman scattering (CARS; **Figure 2B**) and stimulated Raman scattering (SRS; **Figure 2D**), use synchronized pump and Stokes beams to amplify vibrational signals through nonlinear optical interactions (16). These techniques can achieve video rate imaging (microsecond-level pixel residence time, kilohertz frame rate), far exceeding the temporal resolution of spontaneous Raman (31). These advantages have been translated into clinical applications. For instance, stimulated Raman histology (SRH) can perform real-time intraoperative diagnosis of the resection margin of brain tumors (32).

However, the pursuit of speed comes at a cost: traditional SRS and CARS systems typically cover only about 200–300 cm^-^¹ per acquisition, and a wider spectral range needs to be obtained through scanning or multiplexing (16, 30). This limitation hinders the distinction of chemically similar substances (such as protein variants or ECM subtypes) in common imaging regions (such as the CH stretch window), unless hyperspectral or computational methods are applied (33). Therefore, stimulated Raman imaging is particularly practical in visualizing rich substances with unique Raman characteristics, such as lipids rich in CH_2_, and can achieve dynamic tracking of lipid droplets and adipose tissue (34, 35). Dual-frequency SRS performs well in real-time lipid tracking, but comprehensive biochemical analysis requires hyperspectral SRS or spontaneous Raman, and the overlapping signals in the fingerprint region are resolved through computational unmixing (33, 34).

### 2.3 Surface-enhanced Raman scattering

Surface-enhanced Raman scattering (SERS; **Figure 2C**) enhances Raman signals by using rough metal surfaces or plasmonic nanostructures, most commonly gold or silver nanoparticles (36). Two mechanisms mainly contribute to this enhancement. The first is electromagnetic enhancement, which arises from localized surface plasmon resonance on the metal surface. This resonance increases the local electromagnetic field, so molecules located near nanogaps, sharp tips, or other “hot spots” can generate much stronger Raman signals. The second is chemical enhancement, which is associated with charge transfer and electronic interactions between the molecule and the metal surface. Although chemical enhancement is usually weaker than electromagnetic enhancement, it can still affect signal intensity and spectral selectivity (18, 36).

These enhancement mechanisms allow SERS to detect trace molecules that are difficult to measure with spontaneous Raman spectroscopy, making it useful for liquid biopsy, circulating biomarker detection, rare-cell analysis, and single-cell studies (37, 38). However, several issues still limit its routine clinical use, including the reproducibility of SERS substrates, uneven hot-spot distribution, interference from complex body fluids, batch-to-batch variation, and safety concerns related to nanomaterials (39).

### 2.4 Biomedical relevance and comparative advantages

Raman imaging holds a unique position in biomedical research, featuring molecular specificity, sub-micron spatial resolution, and minimal sample preparation requirements (19). Its non-destructive feature allows for repeated volume assessment of living systems and capture of *in-situ* dynamic processes (25, 40). The core advantages include the ability to detect endogenous molecular composition (nucleic acids, proteins, lipids, carbohydrates, and ECMs) without labeling or slicing (23). This is in sharp contrast to traditional histology or mass spectrometry imaging (MSI), which usually requires destructive pretreatment (41, 42). In addition, stimulated Raman methods (such as SRS, **Figure 2D**) have real-time imaging capabilities, filling the gap between static molecular spectra and dynamic biological processes (16).

However, there is an inherent trade-off in Raman imaging: spontaneous Raman trades speed for chemical breadth, while stimulated Raman trades spectral coverage for speed (16). Innovations such as time-domain SRS and multi-path excitation aim to alleviate these trade-offs (22, 30). When compared with other techniques, Raman demonstrates complementary advantages and limitations: Mass spectrometry imaging (MSI), including matrix-assisted laser desorption/ionization (MALDI) and secondary ion mass spectrometry (SIMS), can achieve chemical depth at the omics level, but it is destructive and cannot be used for real-time volume imaging (43). Spatial transcriptomics (ST) can resolve gene expression at near-cellular resolution, but it relies on immobilization, permeation and computational inference, providing indirect rather than direct metabolic or proteomic information (44).

In clinical applications, the choice of Raman modality should depend on the diagnostic question. Spontaneous Raman spectroscopy provides broad spectral coverage and is well suited for detailed biochemical fingerprinting, such as distinguishing proteins, lipids, nucleic acids, carbohydrates, and extracellular matrix components (17, 23, 24). However, its relatively weak signal and slower acquisition make it less suitable for large-area real-time imaging (27). By contrast, coherent Raman techniques, especially SRS, provide much faster imaging and are more suitable for intraoperative or endoscopic scenarios where rapid tissue assessment is required (16, 31, 32). Their limitation is that conventional systems often cover a narrower spectral range, so comprehensive molecular interpretation may require hyperspectral acquisition or computational unmixing (16, 29, 30). SERS offers high sensitivity and is particularly useful for trace biomarker detection in liquid biopsy, but its clinical use depends strongly on reproducible substrates, stable signal enhancement, and standardized sample preparation (36, 39). Therefore, these Raman modalities should be viewed as complementary rather than interchangeable: spontaneous Raman is stronger for detailed molecular profiling, SRS/CARS are stronger for rapid imaging, and SERS is stronger for highly sensitive biomarker detection.

## 3 Applications of Raman spectroscopy in cancer diagnostics

Raman spectroscopy and imaging are garnering increasing attention as versatile tools for interrogating a wide spectrum of malignancies, capitalizing on their intrinsic label-free operation, molecular specificity, and capacity for rapid, even real-time diagnostic readouts (45).

In this section, we synthesize salient advances in Raman-based cancer diagnostics across multiple cancer types. We emphasize applications where the technology provides distinctive value, critically evaluate evidence of diagnostic efficacy, and examine barriers to clinical translation.

### 3.1 Lung cancer

**Figure 3:**
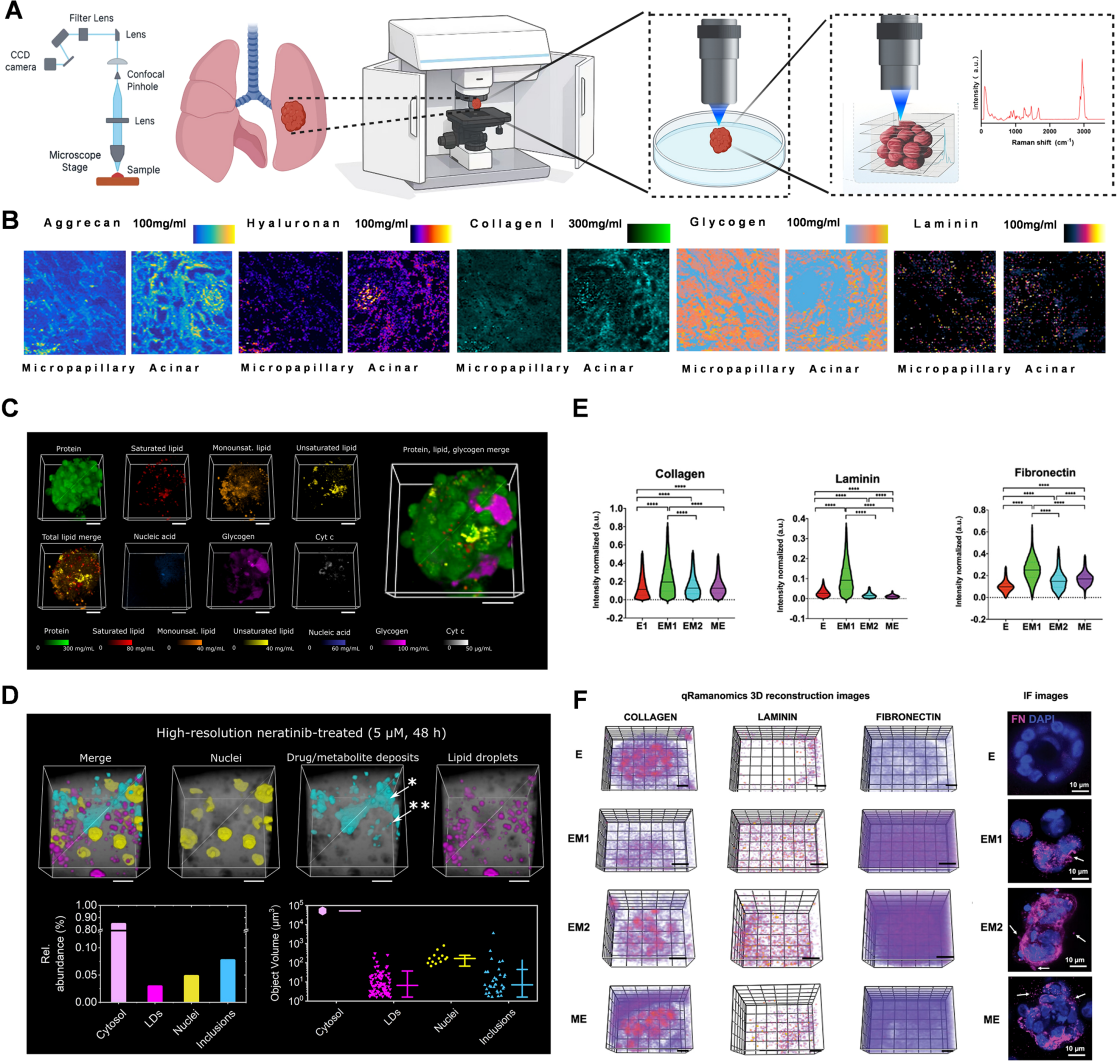
Application of Raman spectroscopy and imaging techniques in cancer and organoid models. **(A)** Spontaneous Raman Imaging workflow: Freshly resected patient specimens are immersed in phosphate-buffered saline (PBS) and directly imaged using a water-immersed lens to generate label-free biochemical profiles in real time. **(B)** Raman imaging of subtypes of lung adenocarcinoma: Revealing differences in extracellular matrix components (aggregator proteoglycans, hyaluronic acid, type I collagen, glycogen, laminin) between micropapillary tumors and alveolar tumors. Image size: 1 mm × 1 mm. Adapted with permission from (104), copyright 2025 Elsevier. **(C)** Raman imaging of liver organoids: It enables multiplex detection of proteins, nucleic acids, glycogen, cytochromic C and lipid subtypes (saturated, monounsaturated, polyunsaturated), and provides quantitative concentrations of biomolecules (milligrams/milliliters or micrograms/milliliters). Scale bars: 50 μm. Adapted with permission from (105), copyright 2023 Elsevier. **(D)** Raman microscopy imaging of liver organoids treated with neratinib: Identification of drug/metabolite deposits, lipid droplets and cell nuclei, demonstrating its application in monitoring treatment responses. Scale bars: 10 μm. Adapted with permission from (105), copyright 2023 Elsevier. **(E)** Raman imaging of human breast epithelial organoids: Shows the composition differences of collagen, laminin and fibronectin in different phenotypes (healthy type E1, epithelial-mesenchymal transition type 1 EM1, type 2 EM2 and mesenchymal transition ME). Statistical analysis was performed using a non-parametric Kruskal–Wallis test (****p < 0.0001). Adapted with permission from (106), copyright 2024 Wiley. **(F)** Three-dimensional Raman imaging of collagen, laminin and fibronectin in breast epithelial organoids, and immunofluorescence staining of fibronectin **(red)** and nuclei stained with 4′,6-diamidino-2-phenylindole (DAPI, blue). Laminin and fibronectin are enriched in EM1 and EM2, highlighting extracellular matrix remodeling during tumor progression. Original internal subpanel labels from the source figures are retained. Scale bars: 10 μm. Adapted with permission from (106), copyright 2024 Wiley.

As shown in **Figure 3A**, freshly resected specimens *in vitro* can be directly imaged with a water immersion objective lens to obtain biochemical distribution maps without the need for exogenous staining (46). Lung cancer tissues show consistent metabolic changes, such as elevated phenylalanine (~1004–1016 cm^-^¹), altered nucleic acid/protein ratios (~1217, ~1335–1356 cm^-^¹), and changes in aromatic residues (~1605 cm^-^¹) (47). As shown in **Figure 3B**, Raman imaging can distinguish between micropapillary and acinar types of lung adenocarcinoma, based on the differences in ECM components (such as aggrecan, hyaluronan, type I collagen, glycogen, laminin). After combining Raman spectral features with principal component analysis–linear discriminant analysis (PCA-LDA), approximately 90% sensitivity was achieved in a multicenter population, which was comparable to gold-standard pathology (48). These findings support the feasibility of Raman spectroscopy for histological differentiation and subtype discrimination in ex vivo lung tissue.

### 3.2 Breast cancer

**Figure 4:**
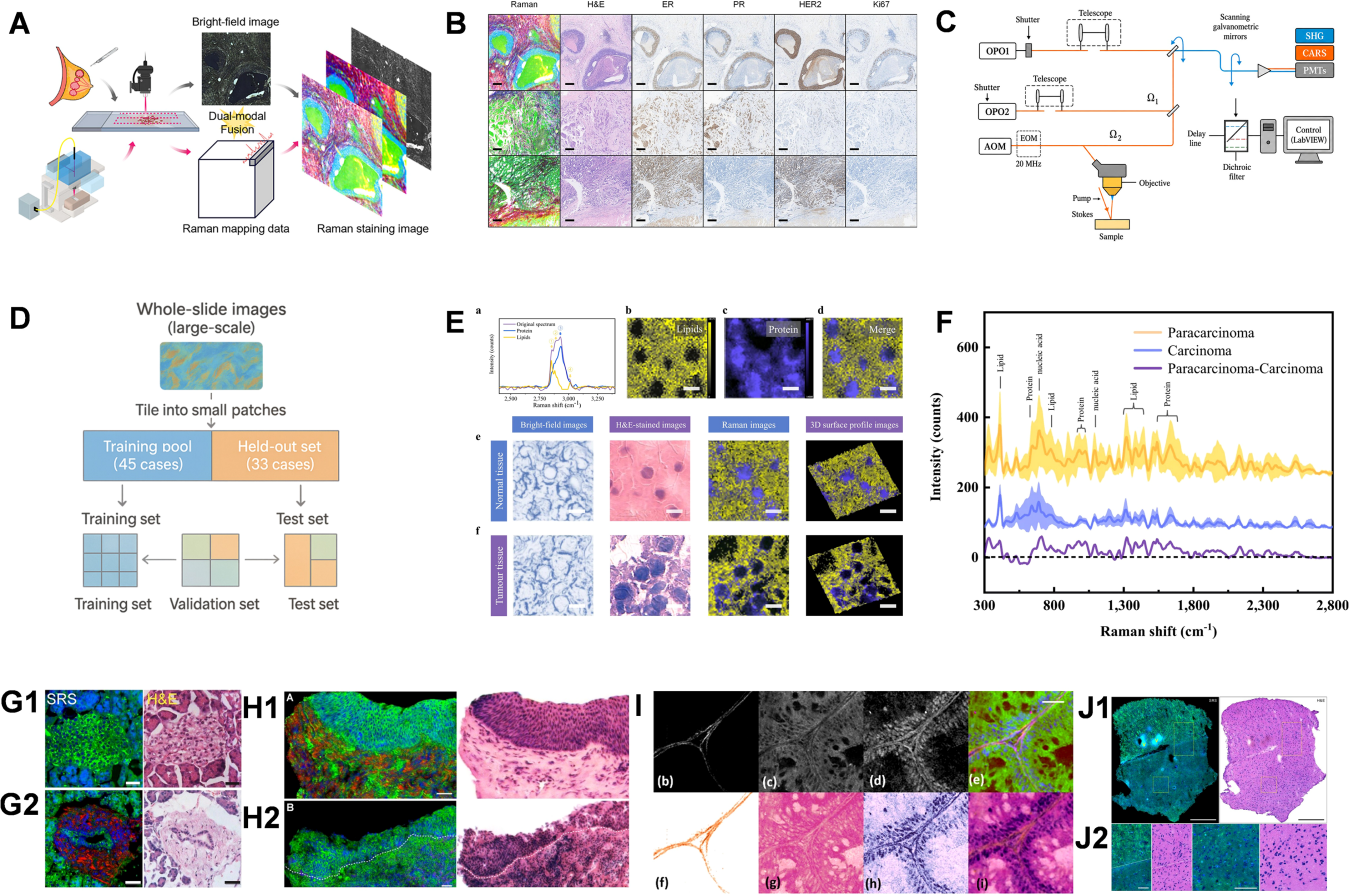
Multimodal Raman imaging for cancer diagnosis. **(A)** Dual-modal fusion of bright-field microscopy and Raman imaging. Adapted with permission from (107), copyright 2024 American Chemical Society. **(B)** Raman staining images of breast tissues of different morphologies and subtypes: carcinoma *in situ*, Luminal A samples, Luminal B samples (HER2 1+ and 2+), and triple-negative samples. The columns display Raman images, H&E staining images and immunohistochemical (ER, PR, HER2, Ki-67) images in sequence. Scale bars: 200 μm. Adapted with permission from (107), copyright 2024 American Chemical Society. **(C)** Schematic diagram of stimulated Raman microscope. **(D)** Diagnostic process based on machine learning. **(E)** Comparison of Raman spectra of liver tissue with reference spectra of proteins and lipids; protein and lipid distribution maps obtained through SMCR. Scale bar: 15 μm. Adapted with permission from (15), copyright 2023 Springer Nature Limited. **(F)** Intraoperative detection of cancer tissues and adjacent tissues was performed using a handheld Raman spectrometer (785 nm). Adapted with permission from (15), copyright 2023 Springer Nature Limited. **(G–J)** Representative stimulated Raman scattering (SRS) images and H&E staining images of the pancreas, larynx, colon and brain tissues, showing key histological features and tumor invasion. Original subpanel labels from the source figures are retained. Scale bars: G1, 50 μm; G2, 50 μm; H1, 30 μm; H2, 100 μm; all panels in I, 30 μm; J1, 500 μm; J2, 100 μm. Adapted with permission from (55), copyright 2021 American Chemical Society. Adapted with permission from (59), copyright 2019 Ivyspring International Publisher. Adapted with permission from (101), copyright 2019 Springer Nature Limited. Adapted with permission from (108), copyright 2016 American Association for Cancer Research.

In breast cancer, Raman-based virtual staining and SRS-based label-free histology have been explored for rapid tissue assessment and automated diagnosis. **Figures 4A**, **B** show representative breast cancer Raman imaging results, including bright-field and Raman image fusion and Raman staining of breast tissue sections. **Figures 4C**, **D** schematically illustrate the stimulated Raman microscopy system and machine-learning-based diagnostic workflow that support Raman/SRS image acquisition and interpretation in this setting (16, 49).

The isolated breast cancer tissues consistently showed enhanced phenylalanine (~1005 cm^-^¹), CH_2_ deformation (~1445 cm^-^¹), and amide I (~1655 cm^-^¹), as well as weakened lipid peaks, reflecting metabolic reprogramming (50). Ex vivo classification models based on convolutional neural networks, linear discriminant analysis (LDA), or support vector machines (SVMs) have often achieved an area under the receiver operating characteristic curve (AUC) above 0.98 (51). As shown in **Figure 4B**, Raman “staining” can distinguish carcinoma *in situ*, Luminal A, Luminal B (HER2 1+/2+), and triple-negative subtypes, and is consistent with hematoxylin and eosin (H&E) staining and immunohistochemistry (IHC) results for estrogen receptor (ER), progesterone receptor (PR), human epidermal growth factor receptor 2 (HER2), and Ki-67 (52–54). This indicates that Raman spectroscopy can provide subtype-sensitive molecular discrimination in ex vivo breast tissue.

### 3.3 Hepatocellular carcinoma

Isolated HCC tissues generally presented nucleic acid-related peaks (785, 1094, 1335, 1578 cm^-^¹) and protein amide I (~1655 cm^-^¹) changes (15). Deep learning models, such as convolutional neural networks (CNNs) and spectral-spatial hybrid models, can achieve a classification accuracy of over 90% in *in vitro* sample validation cohorts (37). As shown in **Figure 4E**, by comparing the protein (2930 cm^-^¹) and lipid (2855/2885/3007 cm^-^¹) based on self-modeling curve resolution (SMCR), the protein/lipid concentration map can be reconstructed and superimposed to present spatial heterogeneity, demonstrating the molecular imaging capability of “near-histology” (15). The same study also showed intraoperative detection of cancerous and adjacent tissues using a handheld Raman spectrometer, supporting the potential of Raman spectroscopy for real-time surgical assessment (**Figure 4F**) (15). These results indicate that Raman spectroscopy may provide molecular and histological information for ex vivo liver tissue assessment.

### 3.4 Pancreatic cancer

At the ex vivo or frozen-section level, Raman and SRS imaging can reconstruct key microanatomical features, such as islets and dense collagen around ducts in normal pancreas, which are consistent with adjacent H&E-stained sections (**Figure 4G**) (55). The reproducible “fingerprint” of pancreatic cancer includes enhanced protein/collagen and relatively reduced lipids/nucleic acids (56). CNN models trained with principal component analysis (PCA) features achieved sensitivity, specificity, and accuracy above 95%, with an AUC close to 0.99 in ex vivo datasets (57). These findings from ex vivo studies provide a spectroscopic and structural basis for future intraoperative margin assessment.

### 3.5 Laryngeal cancer

At *in vitro* or fresh biopsy levels, CNN models based on spontaneous Raman achieved an accuracy of 96.1% and a sensitivity of 95.2% in differentiating benign and malignant conditions (58). As shown in **Figure 4H**, SRS images were highly consistent with H&E-stained sections in laryngeal squamous cell carcinoma, with a Cohen’s kappa coefficient (κ) > 0.90 (59). The 532 nm hybrid Raman fluorescence system also detected cancer-specific peaks such as ~1250, 1400, 1500, and 2900 cm^-^¹ in *in vitro*/early detection (60). These results collectively confirm the high fidelity of Raman spectroscopy and SRS in ex vivo laryngeal assessment.

### 3.6 Colorectal cancer

Miniaturized fiber Raman and multimodal imaging reproduced typical spectral changes (elevated 1004, 1078, 1655 cm^-^¹ and decreased lipid peaks) on isolated colorectal tissues and achieved highly accurate classification (61). As shown in **Figure 4I**, stimulated Raman histology (SRH) combined with second harmonic generation (SHG) and SRS can generate tissue reconstructions close to H&E staining in ex vivo specimens (62). An automated model based on principal component analysis–support vector machine (PCA-SVM) achieved 96.9% accuracy in differentiating normal tissue, adenoma, and adenocarcinoma in 377 ex vivo samples (63).

### 3.7 Gliomas

Raman spectroscopy has achieved tumor/non-tumor discrimination and margin visualization in fresh, frozen, and formalin-fixed, paraffin-embedded (FFPE) brain tissues (12). As shown in **Figure 4J**, SRS and H&E present highly consistent details of the white/gray matter interface, single nuclear shape and infiltrating cells in the *in vitro* sections of oligodendroglioma (grade III) (64, 65). The overall diagnostic accuracy of multi-center handheld devices in ex vivo/intraoperative brain tumor samples is >90% (66). Ex vivo evidence laid the histology-level resolution foundation for its progression to intraoperative real-time navigation.

### 3.8 Gastric cancer

**Figure 5:**
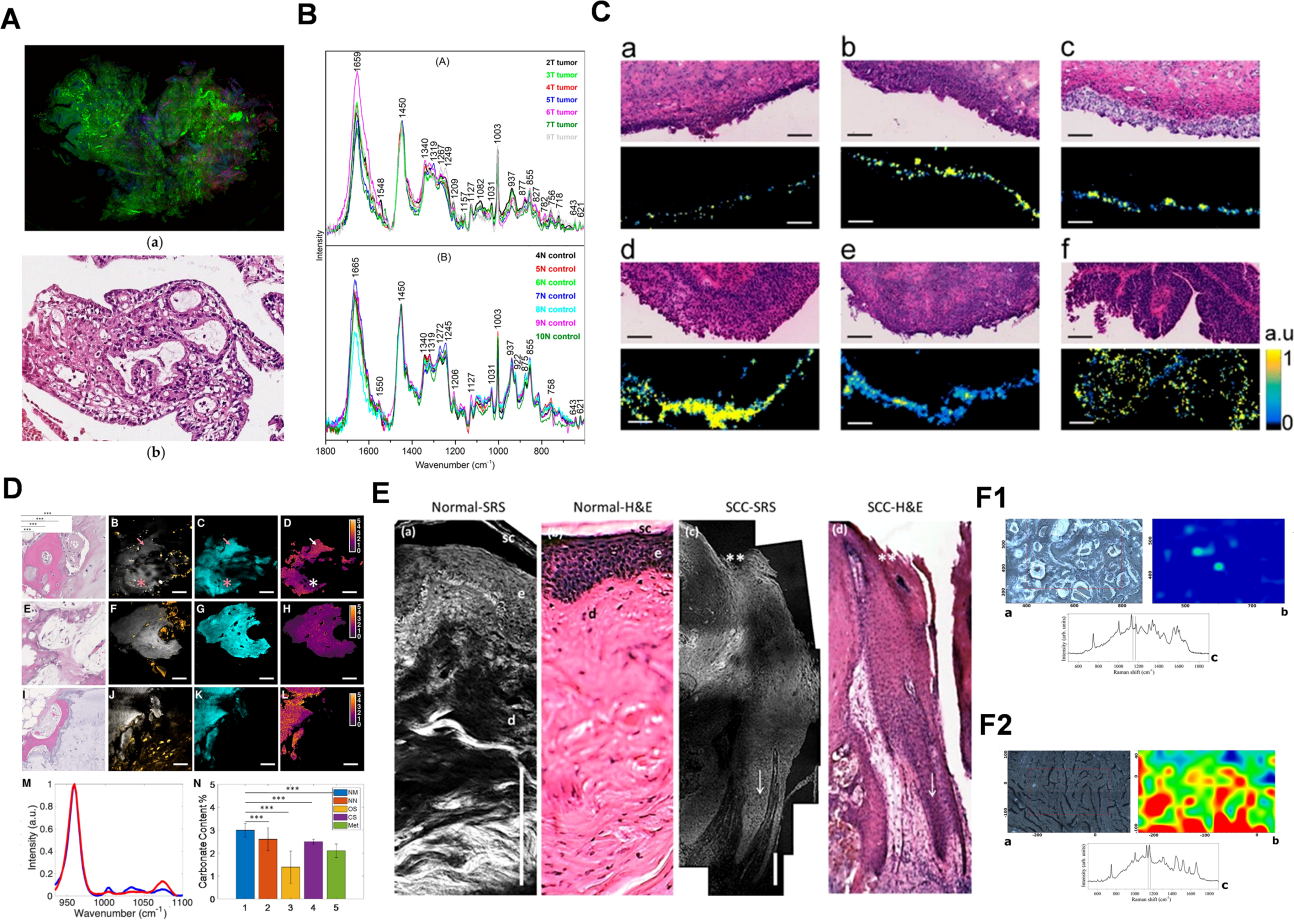
Multimodal Raman and stimulated Raman scattering (SRS) imaging across cancer and tissue models. **(A)** Representative histopathological images of gastric cancer tissues: internal panel a, SRS image; internal panel b, H&E-stained image. The original study reported a pixel resolution of 0.385 μm/pixel for the SRS image and a 20× field of view for the H&E image. Adapted with permission from (109), copyright 2021 MDPI. **(B)** Vertex component analysis (VCA) spectra and corresponding abundance maps of bladder tumor and control tissue. Internal panel A corresponds to bladder tumor tissue, and internal panel B corresponds to control tissue. Adapted with permission from (70), copyright 2023 MDPI. **(C)** The penetration of surface-enhanced Raman scattering (SERS) nanoparticles in bladder cancer. H&E-stained images of normal tissues (internal panels a–c) and cancer tissues (internal panels d–f) (top) and s421-IgG4 Raman images (bottom). Scale bars: 100 μm. Adapted with permission from (73), copyright 2018 American Chemical Society. **(D)** Chemical imaging of bone mineralization. The original internal subpanel labels are retained within this panel. Internal panel A shows H&E staining images of endochondral osteogenesis and bone healing. Internal panels B–D show morphology images (SRS signal at 2930 cm^-^¹; TPF: two-photon fluorescence labeling of nuclei; SHG: second harmonic generation marking collagen), mineralization maps (SRS signal at 960 cm^-^¹), and carbonate content images. Arrows/asterisks mark mature bones and osteoid bones; internal panel E represents the corresponding spectrum. Internal panels F–I show osteosarcoma, and internal panels J–M show chondrosarcoma with neoplastic bone/mineral changes. Internal panel N shows carbonate content (%) in different groups: NM, normal mineralized tissue; NN, normal non-mineralized tissue; OS, osteosarcoma; CS, chondrosarcoma; Met, metastatic foci. Statistical significance in internal panel N was assessed using a two-sided t-test and is indicated by asterisks: ***p < 0.001. In the original study, SRS images were acquired with a field of view of 285 μm × 285 μm (512 × 512 pixels). Adapted with permission from (76), copyright 2022 American Chemical Society. **(E)** SRS mosaic imaging of healthy skin (internal panels a–b) and superficial squamous cell carcinoma (SCC; internal panels c–d). The skin surface is marked; the arrow indicates keratinocyte infiltration. Imaging depth: 300 μm for healthy skin, 1.1 mm for SCC. Scale bars: 100 μm. Adapted with permission from (81), copyright 2013 Wiley Periodicals, Inc. **(F)** Raman chemical chromatogram of thyroid tissue (1156 cm^-^¹ band). (F1) Healthy thyroid tissue: internal panel a, dark-field optical image; internal panel b, Raman spectrum; internal panel c, average spectrum. (F2) Papillary thyroid carcinoma (PTC): internal panel a, dark-field optical image; internal panel b, Raman spectrum; internal panel c, average spectrum. The red square on the right side **(a)** corresponds to the investigated tissue area shown on the left **(b)**. The original study reported Raman maps acquired with a 20 μm step size over a 400 × 300 μm² area; scale bars are shown in the source figure and expressed in μm. Adapted with permission from (84), copyright 2016 Springer Nature Limited.

*In vitro* studies consistently reported robust differences between gastric cancer and normal mucosa at peak positions such as 1003 (phenylalanine), 1250 (amide III), 1445 (CH_2_ deformation), and 1655 cm^-^¹ (amide I) (67). The accuracy rate of traditional machine learning in differentiating gastric adenocarcinoma on isolated samples is over 92% (68). Prospective work in gastric biopsy further shows that deep-learned single-shot femtosecond SRS histology can rapidly distinguish benign from malignant tissue and support biopsy-related decision-making. The paired SRS and H&E images in **Figure 5A** illustrate its histology-like readout, while the reported workflow supports its potential for near-real-time, label-free assessment (69). These findings from ex vivo studies support the potential use of Raman spectroscopy as a histological adjunct in gastric cancer.

### 3.9 Bladder cancer

At the ex vivo level, vertex component analysis (VCA) can extract the basal spectrum from bladder tumors, control tissues and epithelium and generate abundance maps (as shown in **Figure 5B**), revealing the molecular differences among different tissue chambers (70). The overall sensitivity of the systematic review/summary analysis report was 0.91, the specificity was 0.93, and the AUC was 0.97 (including *in vitro* evidence) (71). Typical tissue peaks include ~1002 (phenylalanine), ~1445 (CH_2_), and ~1655 cm^-^¹ (amide I) (72). In addition, SERS nanoparticle imaging has been used to visualize bladder cancer tissue permeability and molecular phenotype, further supporting the potential of Raman-based molecular imaging in bladder cancer (**Figure 5C**) (73, 74). Ex vivo evidence shows that Raman spectroscopy can provide stable discriminative signals across different tissue compartments of bladder tumors.

### 3.10 Malignant bone tumors

Isolated bone tumors showed key changes such as ~960 cm^-^¹ (phosphate ν_1_, mineralization), ~1450 cm^-^¹ (CH_2_), and ~ 1660–1670 cm^-^¹ (amide I) (75). As shown in **Figure 5D**, multimodal SRS, two-photon fluorescence (TPF), and SHG imaging can map mineral deposition, carbonate content, and collagen structure in bone repair, osteosarcoma, and chondrosarcoma samples, supporting differentiation between benign, malignant, and non-tumor lesions (76). These *in vitro* indicators provide information simultaneously in the mineral-organic two-phase (77). From this, a minerality-matrix dual-domain Raman fingerprint suitable for bone tumors was constructed.

### 3.11 Skin cancer

Systematic evaluations have shown that Raman spectroscopy can achieve high classification accuracy for basal cell carcinoma (BCC), squamous cell carcinoma (SCC), and malignant melanoma (MM) in ex vivo samples (78, 79). In the dataset of 731 lesions, the AUC of the spectral model based on a one-dimensional CNN reached 0.909 (covering a large number of ex vivo/quasi-clinical samples) (80). As shown in **Figure 5E**, SRS mosaic images of healthy skin and superficial SCC match the morphology of H&E staining (81). These *in vitro* results demonstrate that Raman spectroscopy can provide molecular profiles close to pathological reading.

### 3.12 Thyroid cancer

Isolated thyroid tissues exhibited malignant characteristics at peak sites such as protein (amide I/III), phenylalanine (1003 cm^-^¹), and nucleic acid. Experimental research reports showed an accuracy of over 90% (82, 83). As shown in **Figure 5F**, the “chemigram” based on 1156 cm^-^¹ clearly distinguishes regional heterogeneity in *in vitro* sections of healthy and papillary thyroid carcinoma (PTC) (84). Explainable artificial intelligence (XAI) methods can correlate spectral segment contributions with pathological biology, enhancing interpretability (82). Ex vivo evidence supports Raman spectroscopy as a decision aid for indeterminate thyroid nodules.

### 3.13 Other cancers: oral and oropharyngeal squamous cell carcinoma, prostate, ovarian/cervical, esophageal cancers, etc.

The isolated tissue/cytological samples of oral squamous cell carcinoma and potential cancerous lesions showed stable differences at isometric peaks of ~1004, ~1445, ~1655 cm^-^¹ (85, 86). The classification accuracy of algorithms such as CNN was over 90%, and the AUC of meta-analysis was approximately 0.93 (87). Ex vivo prostate biopsy or resection specimens showed remodeling of nucleic acids (~785, 1338–1342 cm^-^¹) and lipids (~1445 cm^-^¹), which could be distinguished using PCA-LDA, multilayer perceptron (MLP), or CNN models (88, 89). Ovarian and cervical tumors also achieved a classification performance of over 90% in ex vivo tissue or cytological samples (90, 91). The ex vivo spectra of esophageal cancer showed high discriminative power at peaks such as ~785 and ~1445 cm^-^¹, and the accuracy of PCA-LDA reached 90–97% (92, 93). The common spectral markers across cancer types (~785, 1004, 1338-1342, 1445, 1655 cm^-^¹) support the universality of its “pan-cancer” *in vitro* recognition.

### 3.14 Liquid biopsy and circulating biomarkers

In addition to tissue-based diagnosis, SERS-based liquid biopsy has been explored for non-invasive cancer detection using body fluids, including serum, urine, lavage fluid, and extracellular vesicles. In the liquid biopsy setting, SERS can capture molecular signals from body fluids across multiple cancer types. For example, exosome detection based on bronchoalveolar lavage has been used to identify non-small cell lung cancer (NSCLC). In a population study including 3,551 subjects, serum SERS combined with deep learning achieved an AUC of 0.979–0.988 for cancer discrimination (37, 94). Similar results have also been reported in digestive tract cancers. In colorectal cancer samples, nanostructured SERS achieved an AUC of approximately 0.99 in more than 200 cases, while serum-based multi-cancer detection for gastric cancer reached an AUC of approximately 0.96 (37, 95).

Urine-based SERS has also been investigated for bladder cancer, with reported potential for early detection, recurrence monitoring, and molecular stratification when combined with miRNA panels (96, 97). In thyroid cancer, SERS analysis of fine-needle aspiration (FNA) lavage fluid and serum achieved an AUC of 0.953 and a maximum accuracy of 88% in small-sample studies (98). Microfluidic-SERS platforms have also been used to detect exosome proteins such as CD63, vimentin (VIM), and epithelial cell adhesion molecule (EpCAM) in bone tumors, showing 100% sensitivity and 90% specificity (99). In addition, a cross-cancer SERS-AI platform reported classification accuracy above 96% in multiple body fluid samples, suggesting potential for scalable population-level screening (100).

### 3.15 Comparative synthesis of cancer-specific applications

Across cancer types, Raman-based methods provide different types of diagnostic value rather than a single uniform application. In gliomas and gastrointestinal tumors, the strongest translational rationale is related to rapid tissue classification, endoscopic assessment, and intraoperative decision support. In lung, breast, liver, and pancreatic cancers, Raman spectroscopy is mainly useful for ex vivo molecular histopathology, subtype-sensitive tissue characterization, and margin-related assessment. In bladder, thyroid, skin, and bone tumors, Raman-based methods may serve as adjunctive tools by providing molecular information that complements conventional pathology. SERS-based liquid biopsy differs from tissue imaging and is more closely related to early detection and non-invasive biomarker analysis. However, most applications still require larger patient-level cohorts, external validation, and standardized acquisition protocols before routine clinical use. **Table 1** summarizes these differences across cancer types and liquid-biopsy settings.

**Table 1:** Comparative summary of Raman-based applications across cancer types and liquid biopsy settings.

Cancer type/application	Main sample or setting	Main Raman approach	Main advantage	Current limitation	Representative references
Lung cancer	Fresh or ex vivo lung tissue	Raman spectroscopy/imaging	Detects biochemical and ECM-related differences; supports histological differentiation and subtype discrimination	Mainly ex vivo evidence; larger multicenter validation is still needed	(46–48)
Hepatocellular carcinoma	Ex vivo liver tissue	Raman spectroscopy with deep learning	Provides protein/lipid distribution and molecular histology-like information	Clinical workflow integration and external validation remain limited	(15, 37)
Breast cancer	Ex vivo breast tissue sections	Raman imaging/Raman staining	Supports molecular subtype-sensitive tissue discrimination and comparison with H&E/IHC	Most evidence remains tissue-section based	(49–54)
Pancreatic cancer	Frozen or ex vivo tissue	Raman/SRS imaging	Reconstructs microanatomical features and collagen-rich structures; may support margin assessment	Translation to routine intraoperative use remains early	(55–57)
Laryngeal cancer	Fresh biopsy or ex vivo tissue	Spontaneous Raman/SRS	Provides rapid benign-malignant discrimination and histology-like imaging	Evidence is promising but based on limited clinical cohorts	(58–60)
Colorectal cancer	Ex vivo tissue and endoscopic settings	Fiber Raman/SRH/SRS-SHG	Supports normal-adenoma-cancer classification and potential endoscopic guidance	Needs more prospective in vivo validation	(61–63, 101–103)
Gliomas	Fresh, frozen, or intraoperative brain tissue	SRH/SRS/Raman probes	Strong potential for rapid intraoperative classification and margin assessment	Wider adoption requires standardized imaging and AI validation	(12, 64–66)
Gastric cancer	Biopsy or ex vivo tissue	Raman endoscopy/SRS	Supports rapid biopsy assessment and histology-like imaging	Endoscopic workflow integration still needs validation	(67–69)
Bladder cancer	Resected tissue or urine	Raman imaging/SERS	Provides molecular differences across tissue compartments; urine SERS supports non-invasive testing	SERS substrate and sample-processing standardization are needed	(70–73, 96, 97)
Bone tumors	Ex vivo or intraoperative hard tissue samples	SRS/TPF/SHG multimodal imaging	Maps mineral deposition, carbonate content, and collagen structure	Specialized instrumentation limits broad use	(75–77)
Skin cancer	Ex vivo or superficial tissue samples	Raman spectroscopy/SRS	Useful for molecular discrimination of BCC, SCC, and melanoma	In vivo implementation and clinical workflow need further testing	(78–81)
Thyroid cancer	Ex vivo tissue, FNA lavage, or serum	Raman spectroscopy/SERS	May assist indeterminate thyroid nodule assessment and improve interpretability with XAI	Evidence is still limited by small cohorts	(82–84, 98)
Oral, prostate, ovarian/cervical, and esophageal cancers	Tissue or cytological samples	Raman spectroscopy with ML models	Shows common cancer-related spectral markers and high reported classification performance	Many studies remain retrospective or small-sample	(85–93)
Liquid biopsy	Serum, urine, lavage fluid, extracellular vesicles	SERS/SERS-AI	Enables non-invasive biomarker detection and multi-cancer screening potential	Requires standardized substrates, sample processing, and prospective validation	(37, 94–100)

## 4 Towards *vivo* and intraoperative real-time applications

### 4.1 Endoscopic and fiber-optic probe integration

In the digestive tract endoscopy system, the miniaturized fiber Raman probe that is compatible with conventional colonoscopy can distinguish malignant mucosa from normal mucosa *in vivo*. When combined with PCA-SVM, this approach achieved a diagnostic accuracy above 91% and provided real-time molecular information for biopsy localization and lesion boundary assessment (102, 103). Prospective work on gastric operations further demonstrates that “Raman endoscopy + machine learning” can instantly distinguish between benign and malignant conditions during surgery or treatment and assist in sampling and boundary assessment (69); Meanwhile, the related SRS system design and its parallel images compared with H&E (**Figure 5A**) highlight the potential of SRS to provide histology-like images for real-time, label-free assessment (69, 109). In thoracic surgery and respiratory intervention scenarios, fiber-optic Raman spectroscopy can also provide sub-second-level molecular information for boundary assessment during surgery, with a reported sensitivity of approximately 94% (110). In addition, a hybrid Raman fluorescence platform with a wavelength of 532 nm has identified cancer-related peaks in the upper digestive tract and larynx under *in vivo*/quasi-*in vivo* conditions, supporting early lesion detection and screening applications (60, 111).

### 4.2 Intraoperative microscopy and margin assessment

In neurosurgery, wide-field SRS can generate centimeter-scale tumor probability maps to assist in maximum safe resection; the DeepGlioma workflow based on SRH and artificial intelligence (AI) can provide molecular subtype inference within less than 90 seconds, with an accuracy rate of 93.3% (12, 32). In breast and head and neck surgeries, handheld 785 nm Raman enables real-time non-staining interpretation of cancerous/adjacent cancerous tissues during the operation (**Figure 4F**), while the performance of laryngeal SRS on fresh biopsy is highly consistent with conventional pathology (59, 112). Bone tumor assessment may benefit from multimodal SRS/TPF/SHG imaging, which can map mineral deposition and collagen structure in intraoperative samples and provide both inorganic and organic information for margin assessment (**Figure 5D**) (76). The cryo-sectioned SRS used in pancreatic resection can reconstruct ductal and collagen-rich microstructures (**Figure 4G**), suggesting its potential as an intraoperative margin-assessment tool (56). Similarly, single-point or fiber-optic Raman spectroscopy has shown subsecond lung cancer detection within heterogeneous normal and benign tissue backgrounds, supporting its potential for real-time boundary assessment in thoracic surgery (110).

Summary: These systems bring Raman spectroscopy from the laboratory to the operating room and examination room, providing molecular-level information for real-time decision-making, including sampling, margin assessment, and tumor classification.

## 5 Probing tumor biology: beyond diagnosis

### 5.1 Metabolic imaging and therapy response monitoring

In the field of multi-scale metabolic imaging, liver organoid studies have shown that Raman can simultaneously resolve proteins, nucleic acids, glycogen, cytochromic C, and multiple lipid subtypes, and directly visualize drug/metabolite deposition, lipid droplet formation, and nuclear remodeling after drug treatment (such as neratinib) (105, 113). These findings support dynamic efficacy evaluation in organoid models (**Figures 3C, D**). In respiratory system models, coherent Raman (SRS) is used to track intracellular pharmacokinetics and treatment-induced metabolic reprogramming, providing a quantitative entry point for the study of medication timing and mechanisms (16). In three-dimensional tumor spheres derived from the pancreas, SERS can map the change trajectories of key metabolites such as tryptophan in real time, which is used for comparative evaluation of *in vitro* protocols (114). Early explorations related to immunotherapy also suggest that the dynamics of collagen and lipid metabolism during the treatment of colorectal tumors can be continuously monitored by spectroscopic means, providing a basis for response evaluation and strategy adjustment (115, 116).

### 5.2 Tumor microenvironment and extracellular matrix remodeling

Research on breast epithelial organoids has shown that Raman imaging can analyze the component changes of collagen, laminin and fibronectin among different phenotypes such as E1, EM1, EM2 and ME, and is consistent with immunofluorescence verification (**Figures 3E, F**), focusing on tumor microenvironment and ECM remodeling (117). In line with this, the subtype differences of lung adenocarcinoma can also be distinguished by ECM composition differences, including aggrecan, hyaluronan, type I collagen, glycogen and laminin (**Figure 3B**) (118–120). In pancreatic tissue, the presentation of microenvironmental elements such as dense collagen networks around the ducts by SRS highly corresponds to adjacent H&E sections (**Figure 4G**), jointly demonstrating that this technique can perform *in situ* characterization of tumor microenvironment (TME) and quantify its spatial heterogeneity (55, 121).

## 6 Technical challenges and obstacles to clinical translation

### 6.1 Weak signal and low signal-to-noise ratio

The intrinsic cross-section of spontaneous Raman scattering is only 10^-6^ - 10^-8^, which determines that the signal in biological tissues is very weak, and a low signal-to-noise ratio is commonly seen (122). The endogenous fluorescence and strong scattering of thick tissues will further consume the effective signals, resulting in limited imaging depth. Even if confocal or extended integration time is adopted, the improvement is limited, and it is difficult to accept a longer acquisition time in clinical practice (123). Coherent Raman (such as SRS, CARS) can significantly enhance speed and sensitivity, but often sacrifices spectral bandwidth and resolution, and relies on complex ultrafast laser systems (16). SERS can be enhanced by several orders of magnitude through plasmon amplification, but it requires exogenous metal nanostructures or probes, which in turn brings about hidden concerns regarding biocompatibility, batch-to-batch consistency and regulatory approval (124).

### 6.2 Spectral unmixing and band pattern overlap

The Raman spectrum of biological samples is composed of a large number of partially overlapping vibration bands, and it is difficult to conduct reliable and scalable component identification merely by “peak picking”. The so-called “diagnostic peak” is also often unstable depending on tissue type and operator-dependent factors (125). Therefore, an increasing number of studies adopt the full-spectrum chemometrics approach, achieving unmixing through volume fraction acquisition and matrix factorization (105). For instance, vertex component analysis (VCA) is used for deconvolution of complex spectra (sometimes referred to as “qRamanomics”) (126). The toolchain is also being refined: open-source platforms such as RamanSPy have introduced machine learning and regularization models into preprocessing and large-scale unmixing processes, reducing manual intervention and subjectivity (127). On a larger scale, unlabeled Raman projection tomography can reconstruct the three-dimensional molecular distribution from full-spectrum data, expanding the joint analytical capability of structure and composition (25). However, these methods involve large computational loads and numerous parameters. Currently, most works can only stably unmix a limited number of components (28).

### 6.3 Imaging speed and clinical throughput

In clinical scenarios, especially intraoperative applications, it is necessary for the system to output interpretable molecular comparisons within an acceptable time frame (32). The efficiency of spontaneous Raman limits the speed of point-by-point scanning, resulting in an excessively long time consumption for wide-field imaging (128). SRS can approach video frame rates, but due to the narrow spectral band, the biochemical information it can cover is limited (16, 31). To enhance throughput, studies on forward-turning line scanning, wide-field excitation, and tomographic acquisition, combined with rapid multivariable reconstruction, have demonstrated feasibility in early prospective evaluations (25, 129). To achieve wide deployment, it is also necessary to carry out engineering optimization around the workflow, operator interface and integration with existing surgical/diagnostic equipment (130).

### 6.4 AI-assisted spectral interpretation and clinical decision support

A Raman system intended for clinical use should not only acquire spectra quickly, but also convert them into clear diagnostic information. In many studies, machine learning and deep learning models have been used to classify spectra or Raman-derived images, predict tumor type, or estimate margin status (12, 32, 65, 130–132). However, high accuracy in a single dataset does not always mean that the model can work well in routine clinical practice. Model performance can be affected by sample size, class balance, tissue preparation, laser wavelength, instrument type, and preprocessing steps (125, 127). It may also decrease when the test data come from different patients, centers, or imaging conditions than the training data (133).

Data splitting is another key issue. Raman datasets often contain a large number of spectra from a relatively small number of patients. If spectra from the same patient appear in both the training and testing sets, the model performance may be overestimated. Patient-level splitting and external validation cohorts are therefore important for evaluating real generalizability, especially before clinical testing or prospective validation (133, 134). Biological confounders should also be considered, including inflammation, necrosis, blood contamination, fibrosis, and differences in stromal content, because these factors may change the Raman signal and affect classification (125).

For clinical decision support, the model output should also be interpretable. Instead of only giving a tumor probability, future Raman-AI systems should show which spectral regions contributed to the decision and whether these regions are related to known biological features such as proteins, lipids, nucleic acids, or extracellular matrix components. Such interpretability can help clinicians and pathologists judge whether the model prediction is reasonable (135). Real-time use also requires a complete workflow that integrates spectral acquisition, preprocessing, model inference, and visual display (130–132). At present, many Raman-AI pipelines still remain in the research or early clinical validation stage, and prospective clinical testing is still limited (134, 136).

### 6.5 Standardization, repeatability and regulation

The practices of different institutions in collection, preprocessing and interpretation vary, which directly affects the comparability and reproducibility of the results. The relevant proposals for quantitative vibration microscopy suggest introducing internal standards (such as the OH stretch band of water) for calibration to improve the metrological reliability of the data (137). The community is also developing organizational replicas to conduct cross-laboratory benchmarking tests, but their current application scope is limited (138). There is also uncertainty at the regulatory level. Discussions surrounding the FDA’s regulation of laboratory-developed tests (LDTs) have pointed out the necessity of clarifying the approval path (139). To incorporate Raman diagnosis into routine practice, it is still necessary to design rigorous multi-center clinical validations, establish unified norms, and form an executable registration and supervision plan (136).

## 7 Discussion

Raman spectroscopy has developed into a mature, label-free optical method capable of detecting the molecular composition of biological tissues with high specificity (23). In recent years, advancements in instrument engineering, computational processes, and clinical processes have laid the foundation for real-time oncology diagnosis and gradually introduced deep learning to enhance interpretation capabilities (12). Spontaneous Raman and SRS complement each other’s advantages: The former covers a wider spectral range and is suitable for comprehensive biochemical fingerprint collection; The latter can achieve high-speed imaging on the selected vibration bands and is suitable for rapid screening of a wide field of view (16).

*In vitro* and clinical evidence is constantly accumulating. The combination of Raman or SERS with machine learning has supported non-invasive detection of lung cancer, breast cancer, etc. based on extracellular vesicles (95). In neurosurgery, prospective studies have shown that stimulated Raman histology (SRH) can provide diagnostic results comparable to those of frozen sections in a short period of time and has practical value for intraoperative margin assessment (140). After SRH was combined with modern AI frameworks (convolutional networks and emerging basic models), near-real-time tumor detection and classification have been achieved in early clinical trials (32).

There are still key challenges to be addressed. At present, most AI-based diagnostic models still take morphological alternative indicators as the core, and their generalization ability across cohorts and institutions is limited (133). Conventional H&E staining provides good structural information, but lacks molecular specificity, which has driven the demand for complementary imaging methods that can provide biochemical contrast (141). Raman microscopy can detect the intrinsic vibration signals of proteins, lipids, nucleic acids, sugars and ECM components *in situ*, thereby supplementing this dimension (17). If the reliance on staining and sectioning can be reduced, Raman histology is expected to accelerate diagnostic turnover and better protect tissue integrity (65).

The application scope is expanding rapidly. Raman imaging can map the spatial patterns of metabolic gradients and ECM remodeling *in situ* (24); SERS is also used to track dynamic processes such as epithelial-mesenchymal transition (EMT) (142). In glioma surgery, intraoperative Raman/SRH has demonstrated high diagnostic accuracy (143). In terms of hardware, electron-multiplying charge-coupled device (EMCCD) and scientific complementary metal-oxide-semiconductor (sCMOS) detectors, photon counting schemes, and compact diode laser spectrometers are narrowing the conversion gap (144, 145). MHz-level acquisition systems are approaching true real-time surgical applications (146, 147).

A “division of labor and collaboration” model is taking shape: SRS is responsible for rapid and large-scale intraoperative assessment, while Spontaneous Raman is responsible for more detailed biochemical identification (141). In addition, hybrid platforms of Raman and other optical modes (such as second harmonic or fluorescence lifetime imaging) are being used to provide a more comprehensive characterization of the tumor microenvironment (148). It is worth noting that multi-institutional large model practices (such as the FastGlioma study reported by Nature in 2025) have completed intraoperative tumor detection within 10 seconds, demonstrating the potential of Raman-AI fusion (149). Explainable AI (XAI) for clinical decision-making remains a key link in building doctor trust and meeting regulatory requirements (135).

Among the current barriers to clinical translation, hardware miniaturization and workflow integration are likely to progress first. This is because the technical requirements are relatively clear: the system needs to be smaller, faster, stable, and compatible with existing endoscopic, surgical, or pathology workflows. Recent advances in compact optical design, detector systems, advanced Raman imaging strategies, and MHz-level acquisition have already reduced the gap between laboratory Raman systems and clinically usable devices (144–147). In practical terms, the first clinical applications are more likely to appear in focused scenarios where speed and decision support are more important than full molecular coverage, such as intraoperative margin screening, endoscopic biopsy guidance, and rapid assessment of fresh tissue.

By contrast, AI-assisted interpretation may take longer to mature. Although Raman-AI models have shown promising performance, their reliability depends on whether the model can remain stable across instruments, centers, tissue preparation methods, and patient populations. A model trained on highly controlled spectra may perform less well when applied to samples with inflammation, necrosis, hemorrhage, fibrosis, or variable stromal content. Therefore, future work should move beyond reporting high accuracy in a single dataset and should instead emphasize patient-level validation, external test cohorts, and interpretable spectral features (133, 135). In this regard, XAI is not only a technical add-on, but also a clinical requirement: pathologists and surgeons need to understand whether a model prediction is driven by biologically meaningful signals, such as proteins, lipids, nucleic acids, or extracellular matrix components, rather than by acquisition artifacts or center-specific biases.

The slowest step is likely to be multicenter validation and regulatory implementation. Unlike hardware optimization or algorithm development, this stage requires coordinated protocols across hospitals, standard sample handling, shared quality-control materials, predefined clinical endpoints, and prospective testing in real clinical workflows (134, 136, 150). Different clinical settings may also require different evidence standards. For example, an intraoperative margin tool must prove that it can guide decisions within an acceptable time window, whereas a liquid-biopsy screening platform must demonstrate reproducibility, low false-positive rates, and population-level benefit. Therefore, the translational path of Raman technology will probably be stepwise rather than uniform: compact devices may support near-term use in selected high-value scenarios, AI interpretation will require stronger external validation and explainability, and broad adoption will depend on multicenter clinical evidence and a clear regulatory pathway.

Beyond diagnosis, “spectromics” offer the opportunity to generate biochemical maps with spatial resolution and can be directly compared with emerging spatial omics maps (151). This integrated strategy is expected to connect molecular pathology, computational oncology and precision therapy, promoting a closed loop from mechanism to intervention (152).

In conclusion, Raman spectroscopy has not yet entered routine clinical practice, but miniaturized hardware, scalable AI analysis, and rigorous multi-center validation are jointly enhancing its clinical usability. With the deepening of interdisciplinary cooperation, Raman diagnostic systems are expected to play a greater role in real-time, molecular specificity and patient-centered precision oncology.
